# T-type Calcium Channels in Cancer

**DOI:** 10.3390/cancers11020134

**Published:** 2019-01-23

**Authors:** Lauren Antal, Miguel Martin-Caraballo

**Affiliations:** Department of Pharmaceutical Sciences, School of Pharmacy, University of Maryland Eastern Shore, Princess Anne, MD 21853, USA; lnantal@umes.edu

**Keywords:** T-type Ca^2+^ channel, cancer, proliferation, neuroendocrine differentiation

## Abstract

Although voltage-activated Ca^2+^ channels are a common feature in excitable cells, their expression in cancer tissue is less understood. T-type Ca^2+^ channels are particularly overexpressed in various cancers. Because of their activation profile at membrane potentials close to rest and the generation of a window current, T-type Ca^2+^ channels may regulate a variety of Ca^2+^-dependent cellular processes, including cell proliferation, survival, and differentiation. The expression of T-type Ca^2+^ channels is of special interest as a target for therapeutic interventions.

## 1. Introduction

T-type Ca^2+^ channels can be found in cells throughout the body, including neurons, myocardial cells, and muscle cells [1,2,3]. T-type Ca^2+^ channels allow the influx of extracellular Ca^2+^ at membrane potentials close to rest [4]. They may play an important role in several Ca^2+^-dependent cellular processes, including cell proliferation, survival, and differentiation. T-type Ca^2+^ channels have also been found in cancer cells. T-type Ca^2+^ channel mRNA, protein, and functional expression has been investigated in various cancer cell lines, as well as tumor tissue samples. Pharmacological inhibition or molecular knockdown of T-type Ca^2+^ channel function may be an attractive target in cancer therapy. The aim of this work was to summarize our current knowledge about the distribution and function of T-type Ca^2+^ channels in cancer cells.

## 2. Classification of Voltage-Activated Ca^2+^ Channels

Based on their electrophysiological and pharmacological profiles, voltage-activated Ca^2+^ channels are divided into high voltage-activated (HVA) and low voltage activated (LVA) channels. HVA Ca^2+^ channels are activated by more positive membrane potentials, whereas LVA Ca^2+^ channels are activated near resting membrane potentials and generate inactivating currents [5,6]. Due to their ability to generate tiny currents and their transient activation patterns, LVA Ca^2+^ channels are better known as T-type Ca^2+^ channels and will be the main focus of this review. At least 10 genes that produce the main pore forming α_1_ subunit of the voltage-activated Ca^2+^ channels have been identified. It is believed that gene duplication of the Ca^2+^ channel gene occurred and led to the expression of multiple HVA and LVA Ca^2+^ channels. T-type Ca^2+^ channels are the product of three different genes, including CACNA1G, CACNA1H, and CACNA1I, which encode for the main α-pore forming subunits Ca_v_3.1, Ca_v_3.2 and Ca_v_3.3, respectively [7,8].

In addition to the main α-pore forming subunit, there are also multiple auxiliary subunits that regulate the expression and biophysical properties of voltage-gated Ca^2+^ channels, including α_2_δ, β, and γ [1,3,9]. There are 4 different isoforms of the α_2_δ subunit, α_2_δ_1_, α_2_δ_2_, α_2_δ_3_, and α_2_δ_4_, which are encoded by 4 different genes [10,11,12,13,14]. The α_2_δ auxiliary subunit plays an important role in increasing the amplitude of Ca^2+^ currents [13,14]. Co-expression of α_2_δ and Ca_v_3.1 leads to an increased density of Ca_v_3.1 on the cell membrane compared to Ca_v_3.1 expression alone. Co-expression of both proteins also increases the current density and maximum conductance of voltage-gated Ca^2+^ channels [15,16]. There are 4 isoforms of the β subunit, β_1–4_, which are encoded by different genes [17]. The β subunit’s Beta Interaction Domain (BID) interacts with the Alpha Interaction Domain (AID) on the α_1_ subunit of voltage-gated Ca^2+^ channels and helps enhance trafficking of the α_1_ subunit to the membrane [18,19,20]. However, molecular inhibition of β subunit expression does not affect T-type Ca^2+^ currents [21]. The γ subunit has 8 different isoforms, γ_1–8_, which are encoded by 8 different genes [22]. γ subunits can have an inhibitory effect on Ca^2+^ currents and can alter activation/inactivation kinetics of the Ca^2+^ channels [9,23].

## 3. Biophysical Properties of T-type Ca^2+^ Channels

The α_1_ subunit of T-type Ca^2+^ channels is a 4 × 6 transmembrane structure consisting of 4 domains, with each domain possessing 6 transmembrane segments. Each domain has a voltage-sensing domain, composed of segments S1 to S4, and a pore domain, composed of segments S5 and S6. The S4 segment contains positive gating charges that are necessary for voltage sensitivity. Between the S5 and S6 segments of the pore domain is the reentrant pore, which leads to the channel’s selectivity. Cytoplasmic linkers connect the 4 domains. The length of the cytoplasmic linkers is variable between domains I and II and II and III. However, the cytoplasmic linker between domains III and IV is typically either 53 or 54 amino acid residues in length. The C-terminus of the pore-forming subunit is typically longer, while the N-terminus is typically shorter [24].

T-type Ca^2+^ channels open at a much more negative membrane potential than HVA Ca^2+^ channels, requiring small depolarizations, and are not subject to current rundown [25]. The Ca^2+^ currents generated by T-type channels are transient as a result of voltage-dependent inactivation. Upon repolarization of the membrane, T-type Ca^2+^ channels slowly close, which leads to a slowly deactivating tail current [4,24]. T-type Ca^2+^ channels allow for the permeation of Ba^2+^ into the cell [4,26]. T-type Ca^2+^ channels can have activation and inactivation over similar voltage ranges. They have a window current where they can open, but not inactivate completely, resulting in significant Ca^2+^ entry at membrane potentials near rest [4].

Across different cell types, currents generated by T-type Ca^2+^ channels have similar electrophysiological properties. However, T-type Ca^2+^ channels display differences in their inactivation and how they respond to drugs. Ca_v_3.2 channels are extremely sensitive to inhibition by nickel ions, while Ca_v_3.1 and Ca_v_3.3 channels are much less sensitive [27]. T-type Ca^2+^ channels are also sensitive to temperature. The maximum amplitude for T-type Ca^2+^ currents can be seen at 30 °C [28]. T-type Ca^2+^ channels are also sensitive to pH. An acidic extracellular environment causes the current amplitudes to decrease while an alkaline extracellular environment causes increased current amplitudes [29,30]. pH variations change the activation and inactivation voltage dependence [30]. Several chemicals and toxins alter the function of T-type Ca^2+^ channels, including mibefradil and its derivate NNC 55-0396, kurtoxin derived from scorpion venom, and ProTx-I peptide, a venom toxin isolated from the tarantula [8]. T-type Ca^2+^ channels are less sensitive to inhibition by dihydropyridines.

Modulation of T-type Ca^2+^ channels involves multiple mechanisms [31]. T-type Ca^2+^ channels in mammalian cells can be regulated by protein kinase A (PKA) and protein kinase C (PKC). PKA and PKC activation causes an increase in current amplitudes in all three types of T-type Ca^2+^ channels [32]. Calmodulin plays a role in modulating T-type Ca^2+^ channels through its ability to bind to the helix 2 of the gating brake, which is found in the cytoplasmic linker region between domains I and II. The gating brake in T-type Ca^2+^ channels keep the channel in the closed position at resting membrane potential. A conformational change occurs when calmodulin binds to the gating brake [33].

T-type Ca^2+^ channels play an important role in many physiological processes. They regulate neuronal excitability and firing in excitable cells. T-type Ca^2+^ channels mediate low-threshold Ca^2+^ spikes (LTS) that function as a pacemaker. Increased intracellular Ca^2+^ leads to depolarization of the membrane, triggering action potentials. During deep sleep, the membrane potential of thalamic relay neurons is hyperpolarized and LTS occur, which leads to burst firing [33,34]. T-type Ca^2+^ channels of endocrine tissues regulate hormone secretion [8]. T-type Ca^2+^ channels are also found in smooth muscle cells [35]. The role of T-type Ca^2+^ channels in cancer cells is less understood and is the focus of this review.

Although the molecular expression of T-type Ca^2+^ channel subunits has been assessed in various cancers by PCR and immunoblot analysis, functional channel expression has not been established in many studies (Table 1). The functional expression refers to the presence of T-type Ca^2+^ channels on the membrane as the result of protein trafficking and its ability to allow the flow of extracellular Ca^2+^, which can be assessed by whole cell recordings (Table 1). This is an important consideration when attempting to understand the role of T-type Ca^2+^ channels in cancer progression. Both molecular and functional expression may be regulated by a variety of factors [31,36].

## 4. Expression of T-type Ca^2+^ Channels in Prostate Cancer

The prostate, a gland that is found in male mammals, is responsible for producing a portion of the seminal fluid. The prostate is composed of luminal secretory epithelial cells, basal epithelial cells, and neuroendocrine cells in addition to stromal smooth muscle cells and stem cells. Neuroendocrine cells are rare in the prostate and comprise 1% or less of total cells. Neuroendocrine cells are dispersed among the epithelial cells [56]. Neuroendocrine cells have two different morphologies: open or closed. The open type morphology has extensions that reach the lumen, while the closed type morphology does not. Open and closed neuroendocrine cells have neurite-like processes and cytoplasmic dense core granules. Chromogranin A is the major secretory protein expressed in neuroendocrine cells. Neuroendocrine cells can also express chromogranin B, secretogranin II, serotonin, and neuron-specific enolase (NSE). Some neuroendocrine cells may also release calcitonin, gastrin-releasing peptide, somatostatin, α- human chorionic gonadotropin, a thyroid-stimulating hormone-like peptide, parathyroid hormone-related protein, cholecystokinin, and vascular endothelial growth factor [57,58].

Prostate cancer is the second leading cause of cancer death for male patients of all ages and is first in estimated new cancer cases in males [59]. The growth of prostate cancer cells is androgen-dependent, because most epithelial prostate cancer cells express androgen receptors [60]. Androgen-depletion therapy (ADT) is a recommended treatment of prostate cancer after early detection. However, prolonged ADT leads to the progression of prostate cancer from an androgen-dependent to an androgen-independent form, occurring typically after a few years of therapy [50]. Neuroendocrine differentiation (or trans-differentiation) is a contributing factor to the transition of prostate cancer to an androgen-independent phenotype (also called hormone-refractory prostate cancer). Cell plasticity allows epithelial prostate cancer cells to transition to a neuroendocrine-like phenotype through trans-differentiation [60]. Neuroendocrine and trans-differentiated cells have similar phenotypes. However, there are differences between neuroendocrine cells and cells that have undergone trans-differentiation. Both neuroendocrine cells and trans-differentiated cells lack expression of androgen receptors or prostate specific antigen (PSA) and show low or no proliferation. Therefore, epithelial cells that have undergone trans-differentiation no longer express androgen receptors and are minimally responsive to ADT [50]. Neuroendocrine cells express basal cell markers, tend to be non-aggressive, do not express anti-apoptotic protein B cell lymphoma protein 2 (Bcl-2) or α-methylacyl-CoA racemase (AMACR). Trans-differentiated cells express luminal cell markers, tend to be highly aggressive, and have increased expression of Bcl-2 and AMACR [61]. Neuroendocrine prostate cells can secrete mitogenic factors, which may lead to cancer progression and a poor prognosis. Focal neuroendocrine differentiation occurs where there are groups of neuroendocrine cells surrounded by dividing prostate epithelial cells. The neuroendocrine cells secrete mitogenic factors that stimulate proliferation of the neighboring epithelial cells or neuroendocrine trans-differentiation, which can lead to prostate growth [49].

Several in vitro models involving cell lines have been established to study the progression of prostate cancer to an androgen-independent phenotype. The most widely used cell lines are LNCaP, DU-145, and PC-3 [62]. DU-145 cells are derived from a brain metastatic prostate cancer. These cells are androgen independent and do not express mRNA or protein for androgen receptors or PSA. PC-3 cells are derived from a metastatic prostate cancer to the bones. Like DU-145, PC-3 cells are androgen independent and do not express mRNA or the protein for androgen receptors or PSA. LNCaP cells were isolated from a metastatic prostate cancer to the lymph node. This cell line is androgen dependent and express the mRNA and protein for androgen receptors and PSA [63]. Trans-differentiation of LNCaP cells in vitro can be stimulated using ADT, interleukin-6 (IL-6), elevated intracellular cAMP, or sodium butyrate [52,53,54]. The most commonly used in vivo model involves transplanting human prostate cancer cells into mice [63].

Trans-differentiation of prostate cancer cells in vitro results in the expression of functional T-type Ca^2+^ channels (Table 1). We should point out that LNCaP cells express only the Ca_v_3.2 transcripts with no other channel subunits being detected by PCR analysis [49,53,54]. cAMP-evoked trans-differentiation of LNCaP cells evokes a significant increase in the functional expression of T-type Ca^2+^ channels [49]. Treatment of LNCaP cells with IL-6 increases Ca_v_3.2 protein expression without altering Ca_v_3.2 mRNA levels. IL-6 does not cause an increase in functional channels in the membrane, while co-treatment with IL-6 and the cAMP-inducing agent forskolin (FSK) causes a significant increase in the functional expression of T-type Ca^2+^ channels in the membrane, as assessed by whole cell recordings [53]. The deacetylase inhibitor sodium butyrate also upregulates the functional expression of T-type Ca^2+^ channels in LNCaP cells through transcriptional mechanisms involving upregulation of Ca_v_3.2 mRNA and protein expression [54]. Disruption of androgen receptor signaling evokes significant trans-differentiation of LNCaP cells. Long term culture (≥7 d) of LNCaP cells in androgen-depleted media increases Ca_v_3.2 mRNA and protein expression when compared to non-stimulated cells [49,55]. Similarly, treatment of LNCaP cells with the androgen receptor blocker bicalutamide increases Ca_v_3.2 protein expression without altering mRNA expression. Overall, trans-differentiation of prostate cancer cells under various conditions evokes the expression of T-type Ca^2+^ channels as a result of both transcriptional and post-transcriptional mechanisms [53,54,55]. An increase in Ca_v_3.2 mRNA expression is detected in prostate tumor tissue compared to adjacent normal tissue [64] while increased expression of the membrane protein is reported in benign prostatic hyperplasia or prostate carcinoma tissues in comparison to normal tissue [50].

Increased expression of T-type Ca^2+^ channels in prostate cancer cells undergoing trans-differentiation regulates cell morphology and secretion of mitogenic factors (Table 1). Pharmacological inhibition of T-type Ca^2+^ channel function reduces the number of neurite outgrowths and neurite length in prostate cancer cells undergoing morphological differentiation [49,53,54]. Increased expression of Ca_v_3.2 T-type Ca^2+^ channel subunits leads to increased Ca^2+^-dependent secretion in neuroendocrine differentiated prostate cancer cells, including increased secretion of prostatic acid phosphate (PAP). The overexpressed channels in neuroendocrine prostate cancer may lead to an increased autocrine and paracrine secretion, which may be involved in cancer progression [50]. Downregulation of Ca_v_3.2 expression in LNCaP cells by siRNA results in a significant reduction in cell proliferation [51]. It is unclear whether this effect is a direct result of disrupting cell division or the secretion of mitogenic factors.

The expression of T-type Ca^2+^ channels in prostate cancer cells can be regulated by several factors. Toyota and colleagues determined that the CACNA1G gene, located on chromosome 17q21, is a target for hypermethylation in certain types of cancer, including prostate cancer. The epigenetic modification occurs upstream from CACNA1G and the methylation of CpG islands is closely correlated to transcriptional inactivation of the Ca_v_3.1 gene [65]. The Ca_v_3.2 molecular expression is regulated by the interplay of the transcription factors early growth response 1 (EGR-1) and repressor element 1 silencing transcription factor (REST). EGR-1 is a positive transcriptional regulator of Ca_v_3.2 expression, whereas REST is a negative transcriptional regulator of Ca_v_3.2 expression [52,66]. The hormone ghrelin regulates the expression of the Ca_v_3.1 T-type Ca^2+^ channel subunit in PC3 cells by stimulating Ca_v_3.1 mRNA and protein (Table 1) [37]. Whole-cell patch clamp recordings reveal that ghrelin treated PC-3 cells exhibit functional T-type Ca^2+^ channels [37]. Hydrogen sulfide (H_2_S) modulates T-type Ca^2+^ channel function in prostate cancers cells (Table 1) [52]. H_2_S is formed by the activity of several cellular enzymes, such as cystathionine-γ-lyase and cystathionine-β-synthase. H_2_S production targets ion channels, including T-type Ca^2+^ channels. In trans-differentiated LNCaP cells, exogenous H_2_S donors increase T-type Ca^2+^ currents within 2–5 min. On the other hand, inhibition of the enzymes involved in the endogenous production of H_2_S causes a decrease in the amplitude of T-type Ca^2+^ currents.

Auxiliary subunits can also play an important role in the progression of prostate cancer. α_2_δ_2_ is an auxiliary subunit of voltage-gated Ca^2+^ channels that is expressed in normal and cancerous prostate epithelial glandular acini cells, more on the apical membranes than the basolateral membranes. This auxiliary protein is expressed in LNCaP, DU145 and PC-3 cells [67]. LNCaP cells do not express α_2_δ_1_ or α_2_δ_3_ subunits [67]. The expression of α_2_δ_2_ is upregulated during cancer development. The α_2_δ_2_ subunit is found throughout prostate cancer tissue that has progressed to an intermediate or poorly differentiated state. LNCaP cells with increased expression of α_2_δ_2_ have faster rates of proliferation, indicating that the α_2_δ_2_ subunit enhances cellular proliferation. When LNCaP, DU145, and PC-3 cells are treated with short interference (si)-α_2_δ_2_, there is a decrease in proliferation. Inhibition of α_2_δ_2_ function with gabapentin or pregabalin also decreases cellular proliferation in LNCaP cells. The α_2_δ_2_ subunits contribute to the regulation of Ca^2+^ homeostasis in LNCaP cells. LNCaP-α_2_δ_2_, clones that overexpress α_2_δ_2_, show a greater nuclear factor of activated T-cells (NFAT) activity. Increased expression of α_2_δ_2_ is associated with in vivo tumor development in immunodeficient nude mice. Increased expression of α_2_δ_2_ led to tumors that had more proliferating cells, grew faster and reached larger sizes. Cells that overexpressed α_2_δ_2_ also had more vascular endothelial growth factor (VEGF) production and increased angiogenesis [67].

## 5. Expression of T-type Ca^2+^ Channels in Breast Cancer

Breast cancer is the second leading cause of cancer death for women of all ages and the first leading cause of cancer death for women between the ages of 20–59. It is first in estimated new cancer cases detected in females [59]. There are many factors that increase the risk of developing breast cancer, including early menarche or late menopause, the use of hormone replacement therapy (HRT), fewer pregnancies, and shorter lifetime duration of breastfeeding [68,69,70]. Selective estrogen receptor modulators (SERMs) may be used to reduce the risk of developing breast cancer. Tamoxifen and raloxifene are used in postmenopausal women and they are usual treatments for premenopausal women at high risk of developing breast cancer to reduce the risk of developing estrogen receptor (ER) positive breast cancer [71]. The treatment of breast cancer depends upon the type and stage.

There are multiple cell lines that can be used as in vitro models for breast cancer. The ER positive MCF-7 cell line is one of the most widely used cell lines for breast cancer research. ER expression in MCF-7 cells and ER positive invasive breast cancer in vivo is similar [72]. MCF-7 cells are commonly used in xenografts to study the progression of breast cancer in vivo. MCF-7 cells are human epidermal growth factor receptor 2 (HER2) negative [72,73,74]. The MCF-7 cell line also expresses progestin receptors (PR), androgen receptors, and glucocorticoid receptors [72,75]. Another important feature of MCF-7 breast cancer cells is the expression of Ca_v_3.1 and Ca_v_3.2 T-type Ca^2+^ channel transcripts [40]. T47D is another routinely used cell line that is ER positive, PR positive, HER2 negative. MDA-MB-231 is another commonly used cell line in breast cancer research. This cell line is triple negative, lacking ER, PR, and HER2 [73,74]. While this cell line is invasive in vitro, in vivo studies do not show comparable invasiveness, the invasiveness is appreciably decreased. MDA-MB-453 cells are ER negative, PR negative, and HER2 positive [73,74]. This cell line had poor tumorigenic potential when used in xenografts [73].

In order to better understand the role of T-type Ca^2+^ channels in breast cancer proliferation, Bertolesi and colleagues investigated the effect of pimozide and mibefradil on these channels in MCF-7 cells (Table 1) [39]. Inhibition of T-type Ca^2+^ channel function with pimozide or mibefradil decreases proliferation of MCF-7 cells in culture. A potential issue with this study is that mibefradil and pimozide are not selective for only T-type Ca^2+^ channels. Treatment with mibefradil can also lead to inhibition of L-type Ca^2+^ channels and pimozide can inhibit K^+^ channels [76,77,78]. On the contrary, the L-type Ca^2+^ blocker nifedipine had no effect on MCF-7 cell proliferation [39]. Taylor and colleagues examined the effects of inhibiting the T-type Ca^2+^ channels in three different cell lines, MCF-7, MDA-MB-231, and adriamycin resistant MCF-7, using a new, selective T-type Ca^2+^ channel blocker, NNC-55-0396 and siRNA to knockdown Ca_v_3.1 or Ca_v_3.2 expression [40]. Inhibition of T-type Ca^2+^ channel function or downregulation of Ca_v_3 subunit expression generates a significant reduction in cellular proliferation. This effect is not seen in the non-tumorigenic mammary epithelial cell line MCF-10A. They also examined the level of mRNA expression of Ca_v_3.1 and Ca_v_3.2 in MCF-7 cells. They found that there was no detectable expression in confluent cells, but in non-confluent cells there was increased mRNA expression, suggesting that Ca_v_3 subunit expression may be culture-specific [40].

**Figure 1:**
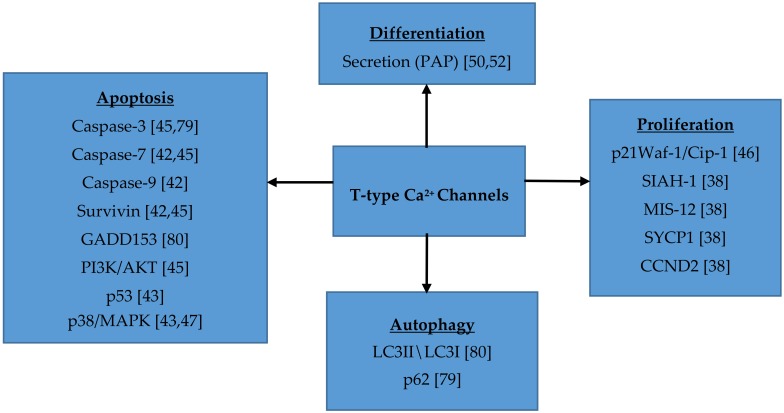
T-type Ca^2+^ channels play a significant role in cellular proliferation, differentiation, autophagy and apoptosis through the activation of various signaling components.

Ohkubo and Yamazaki also investigated the role of Ca_v_3.1 and Ca_v_3.2 in cellular proliferation (Table 1) [38]. They found that siRNA knockdown of Ca_v_3.1 leads to enhanced cellular proliferation in MCF-7 cells (Table 1). Blocking Ca_v_3.1 channels with tarantula toxin, which is a selective T-type Ca^2+^ channel blocker, increases cellular proliferation in a dose-dependent manner, whereas Ca_v_3.1 overexpression leads to decreased cellular proliferation. Knockdown or overexpression of Ca_v_3.2 does not change cellular proliferation. In the non-tumorigenic human breast epithelial cell line MCF-10F, cellular proliferation rates are not affected by either Ca_v_3.1 si-RNA knockdown or Ca_v_3.1 blockade with tarantula toxin. Ca_v_3.1 proteins are typically localized on the cell membranes of MCF-7 cells that display markers of apoptosis, like cell shrinkage, surface blebbing, and chromatic agglutination. In cells that do not display these apoptotic hallmarks, Ca_v_3.1 proteins are typically seen in the cytosol and numerous Ca_v_3.2 proteins can be seen on the cell membrane. Ca_v_3.1 overexpression leads to greater numbers of cells undergoing apoptosis. Cyclophosphamide treatment increases the number of cells that express Ca_v_3.1. Cyclophosphamide induced apoptosis is significantly blocked by si-RNA Ca_v_3.1 expression knockdown. Ca_v_3.1 knockdown shows a decrease in expression of genes related to induction or progression of apoptosis (ERCC2, SIAHI, and GADD34) and an increase in the expression of anti-apoptotic gene STRADB (Figure 1) [38].

In order to better understand Ca^2+^ homeostasis in breast cancer and its role in progression, it is important to look at the auxiliary subunit α_2_δ_3_ [81]. CACNA2D3 is thought to be a tumor suppressor gene in other types of cancer, such as lung cancer and esophageal cancer. In neuroblastomas with a poor prognosis, the expression of CACNA2D3 is downregulated. The downregulation of CACNA2D3 is caused by methylation of the CpG island in the 5′ regulatory sequence of CACNA2D3. Palmieri and colleagues found that breast cancer cell lines MDA-MB-231 and MDA-MB-453 exhibit dense methylation of the CACNA2D3 CpG island while T47D exhibited low-level methylation in comparison to in normal breast epithelial cells. CACNA2D3 mRNA expression in the three hypermethylated cell lines was downregulated. Hypermethylation was also seen in tissue samples from metastases to the CNS. Methylation was examined in primary breast cancer tumors to determine if samples that were positive for methylation were more likely to progress to metastatic breast cancer. Methylation of CpG9 is a biomarker for metastatic relapse to certain sites, especially to the lungs and liver. There was no association between CpG9 methylation and metastases to the skin or lymph nodes [81].

## 6. Expression of Voltage-Activated Ca^2+^ Channels in Ovarian and Other Cancers

Ovarian cancer is the fifth leading cause of death in women of all ages with cancer [59]. Surgery followed by carboplatin/taxane therapy is the standard treatment. Most patients will initially respond to standard therapy, but recurrence can occur, and platinum-resistance may develop [42]. There are several factors that can potentially reduce the risk of developing ovarian cancer, such as the use of oral contraceptives, greater number of pregnancies, tubal ligation, and oophorectomy. Hormone replacement therapy after menopause increases the risk of developing ovarian cancer [82].

Dziegielewska and colleagues examined whether inhibition of T-type Ca^2+^ channels would affect the proliferation and sensitivity of ovarian cancer cells (A2780, A2780Cis, IGROV-1) to platinum chemotherapy (Table 1) [42]. T-type Ca^2+^ channel function was blocked with mibefradil, whereas molecular expression was downregulated with Ca_v_3.1- or Ca_v_3.2-specific siRNA sequences. Inhibition of T-type Ca^2+^ channel function in ovarian cancer cells decreases proliferation and increases apoptosis. It also leads to decreased expression of FOXM1 and BIRC5, which results in reduced expression of the anti-apoptotic gene survivin (Figure 1). Increasing concentrations of mibefradil leads to an increase in the number of cells in the G_1_ phase in a dose-dependent manner for all cell lines tested and a decrease in the number of cells in the S-phase. Treatment of ovarian cancer cells with mibefradil results in lower AKT phosphorylation and nuclear retention of FOXO, which are proteins that decrease BIRC5 expression. Changes in platinum-sensitivity of cells was also investigated. Platinum-resistant cells were pretreated with mibefradil and then carboplatin in vitro. The pretreatment with mibefradil made the previously resistant cells more sensitive to carboplatin. An in vivo model using xenographs in female nude mice shows less tumor growth if the mice were pretreated with mibefradil before receiving carboplatin [42]. Li and colleagues also examined the effects of inhibiting or knocking down the expression of T-type Ca^2+^ channels in ovarian cancer cells [41]. When they examined the expression of T-type Ca^2+^ channels in ovarian cancer cells, they found significantly greater expression of Ca_v_3.1 and Ca_v_3.2 channels in tumor cells compared to normal cells. Cells were treated with mibefradil and siRNA against Ca_v_3.1 and Ca_v_3.2. Additionally, they used NNC-55-0396 to inhibit T-type Ca^2+^ channels. They also found that inhibition or downregulation of T-type Ca^2+^ channel expression results in decreased proliferation in vitro. Like Dziegielewska et al., they also observed that blocking T-type Ca^2+^ channels or knocking down the expression of T-type Ca^2+^ channels leads to a greater number of cells in the G0/G1 phase and reduces the number of cells in the S phase. They also examined the effect of T-type Ca^2+^ channel inhibition with NNC-55-0396 using an in vivo model with nude mice. Treatment with NNC-55-0396 suppresses tumor development, leading to a significantly smaller tumor mass [41].

T-type Ca^2+^ channels play an important part in other types of cancers, such as colon, esophageal, hepatoma, glioma, and melanoma [43,44,46,47,48,83]. In colon cancer cells, Dziegielewska and colleagues found that T-type Ca^2+^ inhibition leads to reduced cell growth and increases p53 dependent apoptosis in cells that express wild type p53 [43]. T-type Ca^2+^ channels were blocked with either mibefradil or TTL1177, which is specific for T-type Ca^2+^ channels. Cells that produce wild type p53 exhibit less growth than cells that produce a mutant p53 isoform. These cells also have a significant increase in the activity of caspase-3/7 (Figure 1). Knockdown of T-type Ca^2+^ channels with siRNA also results in decreased growth. Cells treated with mibefradil have increased expression of cyclin-dependent kinase inhibitor 1A (CDKN1A), which induces cell cycle arrest, and BCL2-binding component 3 (BBC3), a modulator of apoptosis (Figure 1). They found that p38-MAPK is required for mibefradil to inhibit growth and induce apoptosis in colon cancer cells (Figure 1) [43]. Colorectal cancer tissue also shows increased expression of Ca_v_3.1 compared to normal cells [83].

T-type Ca^2+^ channels are abundantly expressed in human melanoma cells (Table 1) [48]. The melanoma cell lines JG, M16 and M28 express the transcripts for the Cav3.1, Cav3.2, and Cav3.3 subunits, whereas melanoma cancer tissue expresses mainly Ca3.1 mRNA. Expression of T-type Ca^2+^ channel subunits can be regulated by exposure to hypoxic conditions. Pharmacological inhibition of T-type Ca^2+^ channel function or gene silencing of channel subunits reduces the viability of melanoma cells, increases the percent of cells in the G-phase and evokes a significant reduction in the number of cells in the S-phase [48].

T-type Ca^2+^ channel expression is observed in different types of brain tumors. According to the American Association of Neurological Surgeons, brain tumors can be classified into two main groups: Primary or metastatic. Primary tumors can be either glial or non-glial and either benign or malignant. 78% of malignant brain tumors are gliomas [84]. The T-type Ca^2+^ channel subunit Ca_v_3.1 is abundantly found in the brain to help regulate neuronal excitability [44,45]. There is increased expression of Ca_v_3.1 mRNA and protein in glioblastoma cell lines and tissue samples (Table 1) [44]. Significant differences in alternative splicing of Cav3.1 subunits are found in normal and cancerous glial tissue. For example, in normal adult brain tissue, isoforms Ca_v_3.1a and Ca_v_3.1bc are expressed more abundantly. In gliomas, the majority of expression is Ca_v_3.1bc and Ca_v_3.1b, and some show expression of a different variant, Ca_v_3.1ac [44]. Valerie and colleagues determined the effect of inhibiting T-type Ca^2+^ channels in glioblastoma cells [45]. They inhibited T-type Ca^2+^ channels by pharmacological means or by knocking down Cav3.1 expression with siRNA. Mibefradil causes a dose-dependent reduction in cellular viability of glioblastoma cells, a loss of clonogenic activity, and increases apoptosis as a result of increased caspase-3/7 activity [45]. Mibefradil treatment leads to lower levels of Mcl-1, Bcl-2, and survivin (Figure 1). Treatment of cells with TTL-1177 also leads to a significant increase in the activity of caspase-3/7. siRNA knockdown of CACNA1G and CACNA1H confirms this activity was related to T-type Ca^2+^ channels. Mibefradil treatment causes a decrease in Akt phosphorylation at Ser473 and Thr308 and a decrease in phosphorylation of Rictor, an mTORC2 subunit [45]. Ernest and colleagues have reported that 68% of the medulloblastoma cell lines express functional T-type Ca^2+^ channels, but there is no evidence of L-type Ca^2+^ channel expression [85].

## 7. Role of T-type Ca^2+^ Channels in Cell Proliferation, Migration, Survival and Differentiation

Ca^2+^ is an important intracellular messenger that helps regulate many different cellular processes, including those that are important to support malignant growth, like proliferation, survival and differentiation (Figure 1) [48]. T-type Ca^2+^ channels show increased expression in several cancer cells compared to normal tissues [64]. Voltage gated Ca^2+^ channels play a role in regulating mitosis. The window current provided by T-type Ca^2+^ channels allows entry of Ca^2+^ into the cell that is necessary for cell cycle progression [48]. Low extracellular Ca^2+^ levels can lead to a decrease in proliferation, causing cells to arrest in the G_1_ phase. Intracellular Ca^2+^ is essential for cellular proliferation. Intracellular depletion of Ca^2+^ can cause decreased DNA and protein synthesis [86]. Oscillatory waves of Ca^2+^, such as those provided by T-type Ca^2+^ channels, are important for cell cycle progression. In smooth muscle cells and fetal cardiomyocytes, functional T-type Ca^2+^ channels increase proliferation. In cancer cells, normal Ca^2+^ signaling is dysregulated and an increased expression of T-type Ca^2+^ channels is often observed [48]. Intracellular Ca^2+^ binds to calmodulin, which leads to its activation. Once Ca^2+^ is bound to calmodulin, it can signal many different processes, including cellular motility and gene transcription. An overexpression of calmodulin can stimulate enhanced cellular proliferation, due to a shorter G_1_ phase [86]. Ca^2+^/calmodulin kinases (CaMK) and calcineurin (CaN, or Ca^2+^/calmodulin-dependent phosphatase) are important downstream targets. Activated calmodulin controls the expression of Ca^2+^-dependent kinases (CDKs), which combine with cyclins to help regulate the cell cycle [87]. Inhibition of T-type Ca^2+^ channels leads to cell cycle arrest in the G0/G1, G1 or G2 phase [41,42,48]. Inhibition of cell proliferation with the T-type Ca^2+^ channel blocker mibefradil may offer new opportunities for tumor treatment. Currently, several pre-clinical and clinical trials are designed to test the efficacy of this compound for the treatment of glioblastoma [80,88].

Expression of T-type Ca^2+^ channels can also regulate cell migration, which may have important implications in the development of metastases. In the fibrosarcoma cell line HT1080, mibefradil causes a concentration-dependent inhibition of cell motility and invasion [79]. Similarly, in glioblastoma U87 cells, mibefradil evokes a significant reduction in cell migration [89]. The proliferative effect of T-type Ca^2+^ channels in glioblastoma cells is altered by endostatin, a C-terminal proteolytic fragment of collagen. Endostatin inhibits T-type Ca^2+^ channel function, resulting in a significant reduction in cell migration [89].

T-type Ca^2+^ channels support malignant growth through cellular survival. Autophagy is a process that helps to maintain cellular homeostasis. Through catabolism, proteins and damaged organelles can be degraded and recycled [90]. In cancer cells, autophagy plays an important role in cellular survival. Basal autophagy maintains homeostasis though degradation of damaged organelles and polyubiquinated, misfolded proteins and when cells undergo stress, autophagy can break down potentially harmful products, and provide nutrients and energy, leading to cell survival [91]. Pharmacological inhibition and molecular knockdown of Ca_v_3.1 and/or Ca_v_3.2 T-type Ca^2+^ channels cause autophagy impairment in melanoma cells (Figure 1). The expression of the autophagy-associated proteins LC3-I/II and p62 increases when T-type Ca^2+^ channel function is inhibited in M16 or JG melanoma cancer cells [90]. This leads to an increase in the protein aggregates that normally would undergo autophagocytosis, resulting in increased apoptosis [92]. As discussed above, pharmacological inhibition and/or molecular knockdown of T-type Ca^2+^ channels can alter cell proliferation by promoting apoptosis. Many different cell lines undergo apoptosis when T-type Ca^2+^ channels are inhibited [37,39,42,43,45]. However, overexpression of Ca_v_3.1 induced apoptosis in MCF-7 cells [38].

## 8. Conclusions

T-type Ca^2+^ channels have been implicated in the progression of various cancers. Ca^2+^ influx via T-type Ca^2+^ channels can activate various signaling pathways. Ca^2+^ activates calmodulin, which in turn controls the expression of many different CDKs that can affect cell cycle regulation and proliferation. Identifying T-type Ca^2+^ channel expression in tumors and characterizing their function may introduce additional treatment alternatives, particularly for patients who are unresponsive to standard therapies. Pharmacological inhibitors of T-type Ca^2+^ channels can induce apoptosis and inhibit proliferation in certain cancer cell lines and also increase sensitivity to certain traditional chemotherapy agents. Thus, T-type Ca^2+^ channels have the potential to become a promising therapeutic target in certain cancers.

## Figures and Tables

**Table 1:** Expression of T-type Ca^2+^ channel subunits in cancer cells and cellular function.

Channel	Sample	mRNA	Protein	Functional Channels	Molecular/Functional Regulation	Cellular Function
Ca_v_3.1	Prostate cancer (PC-3)	+	+	+	Ghrelin ↑ Ca_v_3.1 mRNA, ↑ protein expression	Pharmacological inhibition of channel function promotes apoptosis and decreases proliferation [37]
	Breast cancer (MCF-7)	+	+	ND		Molecular knockdown of Ca_v_3.1 expression decreases proliferation without any effect on apoptosis Overexpression of Ca_v_3.1 promotes apoptosis and decreases proliferation [38]
	Breast cancer (MCF-7)	+	ND	+		Pharmacological inhibition of channel function decreases proliferation [39]
	Breast cancer (MCF-7, MDA-MB-231)	+	+	+	Cell confluency ↓ Ca_v_3.1 mRNA, ↓ protein expression	Pharmacological inhibition of Ca_v_3.1 decreases proliferation [40]
	Retinoblastoma (Y79)	+	ND	+		Pharmacological inhibition of channel function or molecular knockdown of Ca_v_3.1 expression decreases proliferation [39]
	Ovarian cancer (HO8910, A2780)	ND	+	ND		Pharmacological inhibition of channel function or molecular knockdown of Ca_v_3.1 expression decreases proliferation and arrests cells in G0/G1 phase [41]
	Ovarian cancer (A2780, A2780Cis, IGROV-1) ^(1)^	+	ND	ND		Pharmacological inhibition of channel function or molecular knockdown of Ca_v_3.1 expression decreases cell viability, increases apoptosis, arrests cells in G1 and/or G2 phase, decreases survivin and BIRC5 expression, increases sensitivity to carboplatin [42]
	Colon cancer (HCT116 p53wt, HCT116 p53-/-)	+	+	ND		Pharmacological inhibition of channel function or molecular knockdown of Ca_v_3.1 expression decreases proliferation and increases apoptosis [43]
	Glioma (U251N, U563, U87, biopsies)	+	+	+		ND [44]
	Glioblastoma (U251, U87, T98G)	+	ND	ND		Pharmacological inhibition of channel function or molecular knockdown of Ca_v_3.1 expression decreases cell viability and clonogenic potential and increases apoptosis [45]
	Esophageal cancer (TE8)	+	ND	+		Pharmacological inhibition of channel function or molecular knockdown of Ca_v_3.1 expression decreases proliferation without any effect on apoptosis and increases p21^CIP1^ expression [46]
	Hepatocellular carcinoma (SNU449)	+	ND	+		Pharmacological inhibition of channel function, but not molecular knockdown of Ca_v_3.1 expression, decreases proliferation, increases phosphorylated ERK 1/2, and downregulates certain genes [47]
	Melanoma (M28, JG, M16, M29, M9, melanoma tissue samples) ^(1)^	+	ND	+	Hypoxia ↑ Ca_v_3.1 mRNA expression (M16, JG, M28)	Pharmacological inhibition of channel function or molecular knockdown of Ca_v_3.1 expression arrests cells in G1 phase and decreases cell viability [48]
Ca_v_3.2	Prostate cancer (LNCaP)	+	ND	+	Induction of NED with Bt_2_cAMP and IBMX ↑ Ca_v_3.2 mRNA, ↑ functional expression	Pharmacological inhibition of channel function reduces neurite outgrowth [49] Molecular knockdown of Ca_v_3.2 expression inhibits secretion of PAP-prostate acidic phosphatase [50] Molecular knockdown of Ca_v_3.2 expression decreases proliferation [51]
	Prostate cancer (LNCaP)	+	+	+	Induction of NED with Bt_2_cAMP and IBMX or IL-6 increase ↑ Ca_v_3.2 mRNA/protein and functional expressionChannel function can be modulated by H_2_S	Pharmacological inhibition of channel function decreases secretion of PAP-prostate acidic phosphatase [52]
	Prostate cancer (LNCaP)	+	+	+	Induction of NED with IL-6 or sodium butyrate increase ↑ Ca_v_3.2 mRNA/protein and functional expression	Pharmacological inhibition of channel function reduces neurite outgrowth and decreases cell viability [53,54]
	Prostate cancer (LNCaP)	+	+	+	Induction of NED with androgen-depleted media or androgen receptor blocker bicalutamide increase ↑ Ca_v_3.2 mRNA/protein and functional expression	Pharmacological inhibition of channel function decreases cell viability and increases sensitivity to anti-mitotic agents [55]
	Breast cancer (MCF-7)	+	+	ND		Overexpression or molecular knockdown of Ca_v_3.2 expression have no effect on cellular proliferation [38]
	Breast cancer (MCF-7)	+	ND	+		Pharmacological inhibition of channel function decreases proliferation [39]
	Breast cancer (MCF-7, MDA-MB-231)	+	+	+	Cell confluency ↓ Cav3.2 mRNA, ↓ protein expression	Pharmacological inhibition of channel function or molecular knockdown of Ca_v_3.2 expression decreases proliferation [40]
	Retinoblastoma (Y79)	+	ND	+	Differentiation ↓ Cav3.2 mRNA expression	Pharmacological inhibition of channel function or molecular knockdown of Ca_v_3.2 expression decreases proliferation [39]
	Ovarian cancer (HO8910, A2780)	ND	+	ND		Pharmacological inhibition of channel function and molecular knockdown of Ca_v_3.2 expression decreases proliferation and arrests cells in G0/G1 phase [41]
	Ovarian cancer (A2780, A2780Cis, IGROV-1) ^(1)^	+	ND	ND		Pharmacological inhibition of channel function or molecular knockdown of Ca_v_3.2 expression decreases cell viability, increases apoptosis, arrests cells in G1 and/or G2 phase, decreases survivin and BIRC5 expression, increases sensitivity to carboplatin [42]
	Colon cancer (HCT116 p53wt, HCT116 p53-/-)	+	+	ND		Pharmacological inhibition of channel function decreases proliferation and increases apoptosis [43]
	Glioblastoma (U251, U87, T98G)	+	ND	ND		Pharmacological inhibition of channel function or molecular knockdown of Ca_v_3.2 expression decreases cell viability and clonogenic potential, and increases apoptosis [45]
	Esophageal cancer (TE8)	+	ND	+		Pharmacological inhibition of channel function decreases proliferation without any effect on apoptosis and increases p21^CIP1^ expression [46]
	Hepatocellular carcinoma (SNU449)	+	ND	+		Pharmacological inhibition of channel function decreases proliferation, increases phosphorylated ERK 1/2, and downregulates certain genes [47]
	Melanoma (M28, JG) ^(1)^	+	ND	+	Hypoxia ↑ Cav3.2 mRNA expression	Pharmacological inhibition of channel function or molecular knockdown of Ca_v_3.2 expression decreases cell viability and arrests cells in G1 phase [48]
Ca_v_3.3	Ovarian cancer (A2780, A2780Cis, IGROV-1) ^(1)^	+	ND	ND		Pharmacological inhibition of channel function decreases cell viability, increases apoptosis, and arrests cells in the G1 and/or G2 phase, decreases survivin and BIRC5 expression [42]
	Esophageal cancer (TE8) ^(1)^	+	ND	+		Pharmacological inhibition of channel function decreases proliferation without any effect on apoptosis [46]
	Hepatocellular carcinoma (SNU449) ^(1)^	+	ND	+		Pharmacological inhibition of channel function decreases proliferation [47]
	Melanoma (M28, JG) ^(1)^	+	ND	+		Pharmacological inhibition of channel function decreases cell viability and arrests cells in the G1 phase [48]

+ indicates expression of Ca_v_3 channel transcripts by PCR analysis, channel protein by immunoblot analysis, or functional channels as assessed by electrophysiological recordings, ND = not determined, ^(1)^ Detected in combination with other T-type Ca^2+^ channel subunits, ↑: increased ↓: decreased.
